# Concise Review of Corrective Responsive Food Packaging: Recent Advances and Future Prospects

**DOI:** 10.3390/polym18101234

**Published:** 2026-05-18

**Authors:** Hailin Wang, Haowei Lv, Boliang Li, Linyan Deng, Yangyang Wen, Hongyan Li

**Affiliations:** 1 Key Laboratory of Geriatric Nutrition and Health (Beijing Technology and Business University), Ministry of Education, Beijing 100048, China; aoshuwanghailin12@163.com (H.W.); lvhaowei1999@163.com (H.L.); 2 School of Light Industry Science and Engineering, Beijing Technology and Business University, Beijing 100048, China; 18324633564@139.com (B.L.); dliny727@163.com (L.D.)

**Keywords:** food packaging, responsive packaging, active packaging, stimuli-responsive materials, encapsulation technologies

## Abstract

Food packaging constitutes a pivotal enabler within the contemporary food industry, requiring continuous innovation to address evolving challenges. Traditional packaging systems typically provide passive protection, which is inadequate for addressing dynamic microbial shifts and spoilage-induced microenvironmental instabilities. In contrast, corrective responsive food packaging (CRFP) takes a distinct approach through the integration of sensing capabilities and targeted active intervention. Upon detection of specific stimuli, CRFP systems precisely deliver bioactive agents to mitigate food deterioration. This review systematically summarizes recent advances in CRFP technology, offering a comprehensive overview of its core response mechanisms, functional materials, advanced carrier systems, and future research priorities. Special emphasis is given to (i) stimuli-responsive systems, including single-stimulus (pH, enzyme, humidity, temperature, and light) and multi-stimulus-responsive systems, detailing their triggering mechanisms and practical applications; and (ii) functional materials and carriers, exploring their synergistic effects for optimized bioactive release. This review aims to provide a structured framework for the design and implementation of CRFP, facilitating its translation from laboratory to industrial practice and contributing to the development of sustainable and efficient food preservation strategies.

## 1. Introduction

The global food industry plays a pivotal role in the world economy yet continues to face substantial challenges in maintaining the quality and safety of perishable foods while advancing toward sustainable development. Despite concerted efforts to reduce food waste and address world hunger, significant losses persist, driven by microbial degradation, enzymatic reactions, and environmental stressors such as temperature variations and humidity imbalances. According to the Food and Agriculture Organization of the United Nations, approximately one-third of all perishable foods are lost or wasted annually, resulting in an estimated economic loss exceeding USD 100 billion each year. Although these statistics contrast sharply with global hunger trends, food waste remains inextricably linked to food security. In 2024, an estimated 638–720 million people faced hunger, while approximately 2.3 billion people experienced moderate to severe food insecurity [1]. Furthermore, spoilage processes give rise to hazardous conditions, including the proliferation of pathogenic microorganisms and the accumulation of toxic metabolites, which constitute serious public health threats [2,3].

Notably, food spoilage is not a random process but is accompanied by distinct microenvironmental changes. For instance, microbial protein degradation in meat elevates total volatile basic nitrogen (TVB-N) levels and increases pH, while respiration in fruits and vegetables releases carbon dioxide, leading to local acidification. Additionally, spoilage microorganisms secrete hydrolytic enzymes (e.g., cellulases, proteases), which, in combination with fluctuations in relative humidity (RH) and temperature, generate detectable signatures indicative of quality decline [4,5].

Traditional packaging materials, such as polyethylene films and aluminum foils, rely predominantly on physical barrier properties, providing passive protection that fails to adapt dynamically to spoilage-related microenvironmental shifts. Conventional active packaging can extend shelf life by releasing antimicrobials and antioxidants. However, it has a critical limitation: active compounds are released non-selectively. Premature release under high humidity or delayed release during refrigerated storage can lead to suboptimal efficacy or excessive residues, potentially compromising food safety and sensory quality [6,7]. Consequently, the development of responsive packaging systems that can detect spoilage signals, trigger precise responses, and deliver active agents on demand has emerged as a critical strategy for mitigating post-harvest losses and advancing food preservation toward a more targeted and efficient paradigm.

Functional responsive food packaging (RFP) can broadly be categorized into two subtypes based on its objectives [8]: Informative RFP is designed to sense and report spoilage-related changes, translating them into observable or measurable indicators (e.g., colorimetric changes, fluorescence shifts, or electrical signals) without intervening in the spoilage process [9,10]. In contrast, corrective RFP (CRFP) integrates sensing capabilities with active intervention mechanisms, such as the release of antimicrobial or antioxidant agents or the sequestration of ethylene, to actively delay or reverse quality deterioration [11]. The latter type, which directly addresses spoilage through intervention rather than merely detection, is particularly relevant to reducing food waste and preserving nutritional value and thus constitutes the central focus of many practical food preservation applications [2,6].

Given the distinct roles of these subtypes, and considering that CRFP directly targets spoilage through intervention, this review focuses exclusively on this subtype. While several earlier reviews have surveyed responsive food packaging, their scope and depth are often limited in ways that leave critical gaps unaddressed. For instance, comprehensive overviews such as those by Du et al. [2] and Rezaei et al. [6] provide broad catalogs of stimulus–response mechanisms and delivery systems, but they remain largely descriptive, offering little critical assessment of practical bottlenecks or application-specific boundaries. Reviews dedicated to single stimulus types such as pH-responsive systems [5] or enzyme-triggered release [4] naturally focus on one trigger and seldom compare the relative merits or limitations across different stimuli. Likewise, material-centric reviews, including those on hydrogels [12], metal–organic frameworks (MOFs) [13], and lipid nanocarriers [14], deliver detailed physicochemical insights but rarely connect material choice to spoilage-driven design criteria or real-world food matrices. Other recent works discuss responsive packaging in the context of intelligent or active systems [8] yet stop short of proposing structured frameworks that translate accumulated literature into actionable design guidelines. Recognizing these gaps, the present review distinguishes itself through three distinctive contributions. First, beyond describing how each stimulus works, we provide systematic critical assessments of the principal practical bottlenecks and suitable and unsuitable application scenarios for each stimulus type. These critical assessments advance the field beyond simple proof-of-concept demonstrations, providing a more realistic understanding of the practical operational limits. Second, we introduce a comparative material maturity matrix that explicitly weighs technological readiness, regulatory risk, scalability, and cost, which are conspicuously absent from most earlier reviews. This is particularly important for high-performance but safety-uncertain carriers such as MOFs, metal oxide nanoparticles, and photoresponsive nanocomposites. Third, and most importantly, we propose a design-oriented framework that directly links dominant food spoilage pathways (microbial, oxidative, moisture-induced, and multi-factor) with optimal stimuli, compatible carrier architectures, and recommended release kinetics. By bridging mechanistic principles with material and encapsulation choices, this framework is intended to serve as a practical guide for researchers and food technologists who need to select or engineer corrective responsive packaging for real-world preservation challenges. Collectively, these features transform our review from a descriptive catalog into a critical, decision-enabling resource that advances the field toward rational design and more targeted research efforts.

## 2. Methodology

**Figure 1:**
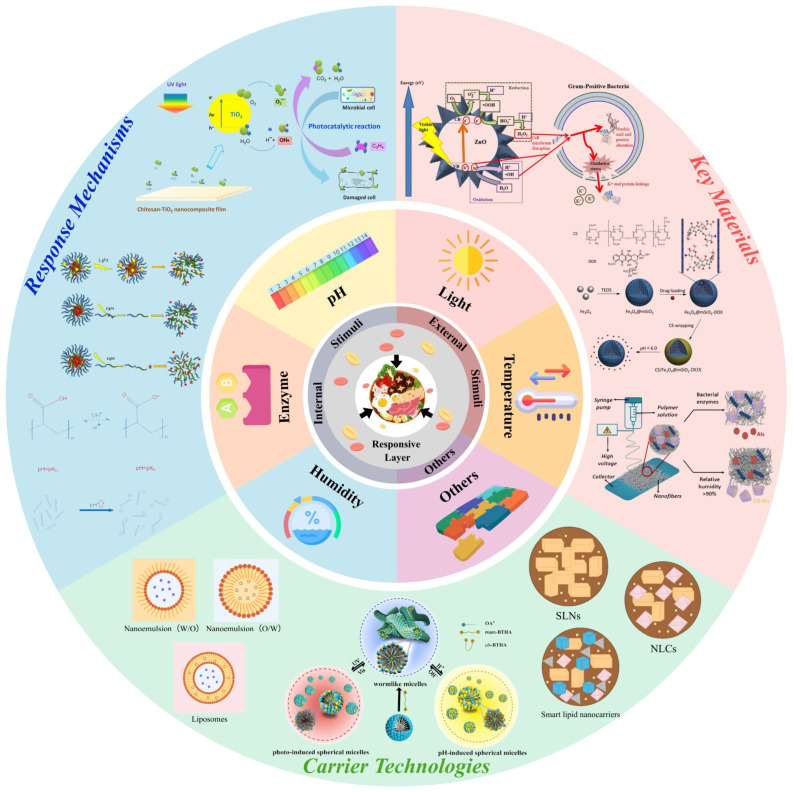
Schematic diagram of responsive food packaging: classification, response mechanisms, and key materials/carrier technologies.

This review aims to critically evaluate recent advances in CRFP, with a particular focus on stimulus-response mechanisms, functional materials, and encapsulation technologies. The purpose is threefold: (i) to systematically categorize and describe the dominant single- and multi-stimulus-responsive systems (pH, enzyme, humidity, temperature, light, and multi-stimulus) and their underlying triggering mechanisms; (ii) to critically assess the practical limitations, scalability, and real-world feasibility of each system, moving beyond descriptive summaries; and (iii) to extract actionable design guidelines by linking food spoilage pathways to appropriate trigger stimuli, key materials and carrier technologies. The scope of this review encompasses all major types of CRFP systems that actively intervene in the spoilage process through the responsive release of antimicrobials, antioxidants, or other bioactive agents. It specifically excludes informative (indicator-type) responsive packaging, which reports spoilage without intervention. The review covers: (1) pH-, enzyme-, humidity-, temperature-, light-, and multi-stimulus-responsive systems, detailing their chemical and physical response mechanisms; (2) key material platforms, including natural biopolymers (chitosan, starch, cellulose), synthetic food-contact polymers (e.g., polylactic acid (PLA), poly(butylene adipate terephthalate) (PBAT), poly(ethylene glycol) (PEG), poly(ε-caprolactone) (PCL)), advanced carriers (mesoporous silica nanoparticles, MOFs, metal oxides), and encapsulation systems (micelles, hydrogels, lipid-based nanocarriers) (Figure 1); and (3) practical aspects such as scalability, regulatory status, safety, and cost level, which are often overlooked in conventional reviews. The function of this review is to provide a structured, critical, and design-oriented resource that helps researchers and food technologists select or engineer appropriate CRFP systems for specific food preservation challenges. The intent is to bridge the gap between proof-of-concept demonstrations and real-world application through comparative assessments of technological maturity, identifying suitable and unsuitable application scenarios, and proposing a design matrix.

The literature included in this review was selected based on predefined inclusion criteria: (i) relevance to corrective (active intervention) responsive food packaging; (ii) publication in peer-reviewed scientific journals or reputable books; (iii) availability of experimental data, mechanistic insights, or comparative analysis; and (iv) a primary focus on recent advances (2020–2026) to ensure up-to-date coverage, although seminal older works were also included where necessary to establish fundamental principles. Exclusion criteria included studies focused solely on informative (indicator) packaging and works lacking sufficient experimental or analytical detail. The literature search was conducted using major scientific databases, including Web of Science, Scopus, PubMed, and Google Scholar, employing keyword combinations such as “responsive food packaging”, “active packaging”, “controlled release”, “stimuli-responsive materials”, “pH-responsive”, “enzyme-responsive”, “humidity-responsive”, “temperature-responsive”, “light-responsive”, “multi-stimulus food packaging”, “chitosan packaging”, “MOF-based food packaging”, “micelles food packaging”, and “hydrogels food preservation”. The search was performed iteratively, with additional references identified from the bibliographies of retrieved articles. The selection process involved an initial screening of titles and abstracts, followed by full-text evaluation to confirm relevance to the defined scope. Priority was given to studies that provided quantitative performance data, comparative analysis across different stimuli or materials, or critical discussion of practical limitations and regulatory aspects. Non-peer-reviewed sources (e.g., preprints, conference abstracts without full data, industrial white papers) were generally excluded unless they offered unique insights not available elsewhere.

## 3. Response Mechanisms and Material Systems

### 3.1. pH-Response System

**Figure 2:**
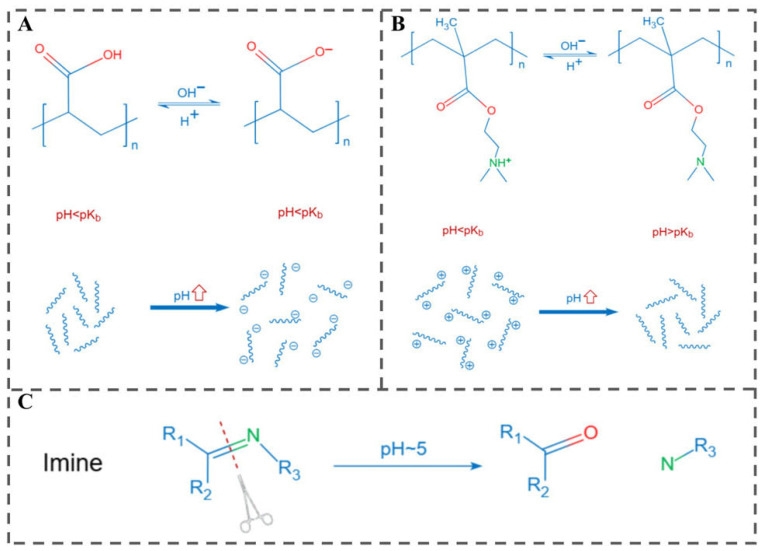
Examples of pH-responsive polymers. (**A**) Polyacids: poly (acrylic acid); (**B**) polybase: poly (N,N-dimethylaminoethyl methacrylate); (**C**) polymers with acid/base-labile linkages: imine.

Food quality deterioration is accompanied by significant changes in pH, rendering pH a key parameter for monitoring the freshness of perishable foods [3]. Due to their operational simplicity and tunable response range, pH-responsive systems have emerged as a focal area in responsive packaging research. Two key pH-changing processes occur during food storage. One is acidification, caused by the buildup of acidic metabolites such as oxalic acid and carbonic acid. The other is alkalinization, driven by TVB-N released from spoiled muscle foods or alkaline metabolites produced by some fungi [2]. These variations serve as stimuli for designing pH-responsive materials, primarily through three mechanisms (Figure 2): (1) Polyacids. Polyacidic pH-responsive polymers, bearing functional groups such as carboxylic, sulfonic, phosphoric or boronic acid groups, accept protons at low pH and release them at neutral/alkaline pH, leading to transitions in solubility or swelling behavior. (2) Polybases (Polycations). Polycationic systems containing tertiary amine, morpholino, pyridine or imidazole moieties become protonated and positively charged at low or neutral pH, subsequently deprotonating and losing charge at higher pH. (3) Polymers with acid/base-labile linkages: Polymers incorporating pH-cleavable linkages (e.g., acetal, hydrazone, imine, ester) undergo selective bond scission under specific acidic or basic conditions, triggering controlled degradation or payload release [5].

#### 3.1.1. Polyacids

Weakly acidic polymers function as pH-responsive materials. Polyacids bearing carboxylic acid groups with pKa values around 5–6 are representative weak polyacids. Among these, poly(acrylic acid) (PAA) and poly(methacrylic acid) (PMAAc) are the most extensively investigated pH-responsive polyacids. At low pH, their carboxylic groups are protonated, whereas at high pH, they deprotonate. Consequently, these polyacids transform into polyelectrolytes at high pH, exhibiting electrostatic repulsion among molecular chains. This effect, combined with hydrophobic interactions, governs processes such as molecular chain precipitation/solubilization, hydrogel swelling/deswelling, and surface hydrophobic/hydrophilic transitions [15]. Hu et al. fabricated a pH-responsive polyvinyl alcohol/poly(acrylic acid) (PVA/PAA) antibacterial film loaded with aminoethyl-phloretin (AEP) [16], whose pH-dependent release relies on the protonation/deprotonation of carboxylic groups in PAA. During pork storage, spoilage microorganisms proliferate and degrade meat proteins, producing a large amount of alkaline TVB-N. This causes a continuous pH increase at the food-packaging interface, which acts as an endogenous stimulus that triggers the material response. Specifically, the carboxyl groups are deprotonated under alkaline conditions, and the electrostatic repulsion between negatively charged chains leads to significant swelling of the film network and realizes the on-demand accelerated release of AEP in response to the degree of pork spoilage. PVA/PAA-AEP films showed AEP content-dependent antioxidant and antimicrobial properties and prolonged pork shelf life by 4 days at 25 °C, demonstrating promising potential for responsive food packaging.

#### 3.1.2. Polybases

Weak polybases function as pH-responsive polymers, exhibiting ionization/deionization transitions typically within a pH range of approximately 7–11. Amine groups in their side chains accept protons under low pH to form polyelectrolytes and release protons in basic environments. Among basic polymers, those based on tertiary amine methacrylates—including poly(dimethylaminoethyl methacrylate) (PDMA), poly[(2-diethylamino)ethyl methacrylate] (PDEA), and poly[(2-diisopropylamino)ethyl methacrylate] (PDPA)—are the most commonly preferred [17]. Polymers containing pyridine or imidazole are distinguished by their basic nitrogen atoms within cyclic structures, enabling protonation/deprotonation reactions in response to specific pH changes [18]. Their inherent pH sensitivity makes them valuable building blocks for various pH-responsive nanostructures. Chitosan is a natural, positively charged polysaccharide and a paradigmatic pH-responsive weak polybase. Its pH sensitivity, due to a high density of amino groups, manifests as rapid dissolution under low pH, insolubility at elevated pH, and pH-dependent swelling driven by amine group protonation under acidic conditions [5]. These properties have led to extensive research on chitosan. Ezati and Rhim fabricated a chitosan-based pH-responsive color-changing film incorporating alizarin for responsive packaging applications [19]. The composite film exhibited a vivid color change from slightly yellow to purple in response to pH changes between 4 and 10. Additionally, it showed strong antibacterial activity against *E. coli* and *L. monocytogenes*, along with potent antioxidant activity. Additionally, Pola et al. developed pH-responsive nanoparticles based on poly(D,L-lactide-co-glycolide) (PLGA) and chitosan for the controlled release of trans-cinnamaldehyde (TCIN) [20].

#### 3.1.3. Polymers with Acid/Base-Labile Linkages

Polymers containing acid- or base-labile linkages undergo specific chemical reactions in response to pH changes, primarily through bond cleavage and degradation [21]. Acid-labile bonds, such as hydrazone, reversibly cleave under acidic conditions, leading to polymer chain hydrolysis, degradation, or aggregate dissociation [22]. Beyond hydrazone, other commonly utilized acid-sensitive bonds include acetal, orthoester, imine, and vinylether [23]. In food preservation, reversible imine bonds have been used as a strategy to develop pH-responsive systems. Heras-Mozos et al. fabricated chitosan-based structures cross-linked with citraldehyde or benzaldehyde, which remained stable in buffers at pH 7 and 4 but rapidly dissolved at pH 3 [24]. Furthermore, grafting trans-2-hexenal and citral onto amino-rich chitosan films via imine bond formation enhanced pineapple preservation [25]. The acidic juice exudate released from fresh-cut pineapple during cold storage acts as the endogenous stimulus, which triggers the hydrolysis of imine bonds on the chitosan matrix, thereby realizing the on-demand release of volatile aldehydes into the package headspace. The released aldehydes exhibited potent antifungal and antibacterial activities against the dominant spoilage microorganisms in pineapple (including wild yeast, molds and *E. coli*) and also effectively delayed enzymatic browning of the fruit, ultimately extending the shelf life of refrigerated fresh-cut pineapple.

Despite the widespread investigation of pH-responsive systems, most studies have been validated under extreme pH conditions (e.g., pH 3.0 or 11.0), whereas real food spoilage typically involves subtle pH shifts of only 0.5–1.5 units. This discrepancy leads to a risk of either false triggers or insensitivity in practice [26,27]. Moreover, the scalability of many pH-responsive carriers remains questionable. For instance, chitosan-based films are easily prepared by solvent casting, and chitosan’s natural origin, biocompatibility, and established GRAS (Generally Recognized As Safe) status make it particularly suitable for food applications [28,29]. In contrast, pH-responsive systems relying on synthetic labile linkages (e.g., hydrazone or imine) often require multi-step synthesis and organic solvents, which raise cost and regulatory concerns for food contact [30]. pH-responsive packaging is best suited for foods with a near-neutral initial pH and a clear acidifying or alkalinizing spoilage pattern. Fresh meat, for instance, undergoes alkalinization due to volatile base nitrogen accumulation [16], while pasteurized milk acidifies through lactic acid production [31]. It is unsuitable for inherently low-pH foods such as yogurt or sauerkraut or products with high buffering capacity (e.g., phosphate-injected meat), where pH signals are masked or dampened. For processed meats, phosphates are commonly added as water-binding agents [32], and such phosphate buffers can effectively absorb protons or hydroxyl ions generated by microbial metabolism, suppressing pH fluctuations until spoilage has already progressed substantially. Successful pH-responsive packaging requires carefully matching the system’s response range to the specific spoilage chemistry of the target food [26]. Rather than relying on universal systems, it is more practical to develop application-specific designs tailored to particular food categories and their characteristic spoilage patterns. pH-responsive packaging should be regarded not as a panacea but as a specialized tool best used when pH reliably and detectably marks spoilage.

### 3.2. Enzyme-Response System

**Figure 3:**
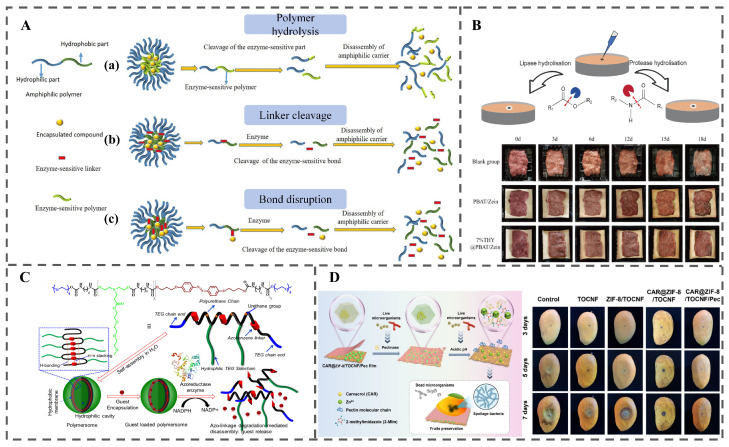
Core aspects and applications of enzyme-responsive systems. (**A**) Mechanisms of enzyme-triggered bioactive release [6]; (**B**) lipase/protease-driven enzyme-response, with chilled mutton preservation photos [33]; (**C**) enzyme-responsive azobenzene-based polyurethane [34]; (**D**) mechanisms of the ZIF-8/TOCNF carrier modified with a pectin “gatekeeper” for pH/enzyme dual-responsive release of CAR, accompanied by application photographs [35].

Microbial spoilage in foods like fresh produce, meats, and bread is often mediated by enzymes such as cellulases, proteases, pectinases, and amylases. Utilizing these enzymes as stimuli to hydrolyze carrier materials and trigger functional molecule release offers a promising strategy for RFP [2]. Enzyme-triggered release operates through three primary mechanisms (Figure 3A) [6]: (1) Hydrolysis of enzyme-sensitive polymers. Carriers with enzyme-labile polymeric backbones undergo site-specific hydrolysis upon exposure to target enzymes, disrupting structural integrity and enabling controlled release. (2) Cleavage of enzyme-sensitive linkers. Amphiphilic polymers with enzyme-sensitive linkers undergo linker cleavage by target enzymes, disrupting intermolecular forces and causing micellar disaggregation for bioactive release. (3) Disruption of enzyme-sensitive bonds. Encapsulated bioactives conjugated via enzyme-sensitive bonds are released upon specific enzymatic cleavage of these linkages.

#### 3.2.1. Hydrolysis of Enzyme-Sensitive Polymers

Enzyme-responsive polymer carriers exploit the specificity of microbial enzymes for targeted bioactive release. Carriers with enzyme-labile polymeric backbones undergo selective enzymatic hydrolysis, triggered by specific enzymes. This mechanism enables precise cargo release while maintaining structural integrity under non-target conditions. Jiang et al. exemplified this approach with enzyme-responsive thymol (THY)@PBAT/Zein fibers. These carriers combine PBAT, containing ester bonds susceptible to microbial lipases, with zein harboring peptide bonds vulnerable to proteases [33]. Upon exposure to bacterial enzymes (Figure 3B), enzymatic hydrolysis disrupts the carrier structure, triggering controlled release of encapsulated THY. This system effectively inhibited microbial growth and extended the shelf life of chilled mutton by 6 days, demonstrating the practical application of enzyme-responsive packaging technologies.

#### 3.2.2. Cleavage of Enzyme-Sensitive Linkers Within the Polymer

Amphiphilic polymers comprise hydrophilic and hydrophobic segments connected via enzyme-sensitive linkers. Upon exposure to target enzymes, these linkers undergo specific cleavage, which disrupts the intermolecular forces stabilizing the micelles. This disruption causes micellar disaggregation, thereby enabling the controlled release of encapsulated bioactives. Kolay et al. engineered an azobenzene-based amphiphilic polyurethane (PU_mAZO_) that self-assembles into polymersomes, with its backbone incorporating azobenzene linkers as enzyme-sensitive moieties [34]. In the presence of azoreductase (a tumor-overexpressed enzyme) and the coenzyme NADPH, these azobenzene linkers undergo specific enzymatic cleavage via azo bond reduction. This cleavage event disrupts the polymer’s structural integrity, triggering polymersome disassembly, which in turn enables controlled release of encapsulated bioactives such as doxorubicin (Figure 3C).

#### 3.2.3. Disruption of Enzyme-Sensitive Bonds Between Encapsulated Bioactives and the Carrier

In this category, encapsulated bioactives are conjugated to the carrier via enzyme-sensitive bonds. Upon exposure to target enzymes, these linkages undergo specific cleavage, directly releasing the bioactives from the carrier. For example, a Carvacrol (CAR)@ZIF-8/TOCNF/Pec composite film employs pectin as a “gatekeeper” that is electrostatically adsorbed onto the surface of the ZIF-8/TOCNF carrier (ZIF-8: zeolitic imidazolate framework-8; TOCNF: TEMPO-oxidized cellulose nanofibers), where pectin forms enzyme-sensitive linkages with the carrier system [35]. When exposed to pectinase secreted by spoilage microorganisms during fruit storage, these pectin-based linkages are specifically cleaved. This cleavage disrupts the protective pectin “gatekeeper” coating, directly facilitating the dissociation of encapsulated CAR from the ZIF-8/TOCNF carrier and triggering its controlled release. The released CAR exerts strong antibacterial and antifungal activities, effectively inhibiting the growth of spoilage microorganisms on fruit surfaces, thereby delaying quality deterioration and extending the shelf life of perishable fresh fruits (Figure 3D).

The specificity of enzyme-substrate interactions necessitates careful consideration of food-susceptible microorganisms and their secreted enzyme profiles in the design of enzyme-responsive systems. Bacteria secrete enzymes like phospholipases, lipases, proteases, hydrolases, nucleases, and glycosidases, while fungi secrete pectinases, cellulases, and ligninases [4]. Many of these enzymes can degrade food matrix components and consequently trigger enzymatic responses. Concomitantly, the design of such responsive materials should integrate both the specific substrates recognized by target enzymes and critical external environmental factors (e.g., temperature and moisture) that modulate enzymatic hydrolysis. For example, enzymatic activity peaks at optimal temperatures, maximizing hydrolysis [36]. Moisture activity typically enhances enzyme activity and polymer hydrolysis in a non-linear manner [37]. The translational potential of enzyme-responsive systems is further influenced by material processability and regulatory accessibility. Systems based on natural enzyme-sensitive polymers (e.g., starch, zein, pectin) are relatively straightforward to process using established techniques such as electrospinning or solvent casting, and their components enjoy GRAS or food-contact status. In contrast, designs employing synthetic enzyme-sensitive linkers often require multi-step organic synthesis, raising manufacturing costs and regulatory barriers. In terms of practical feasibility, enzyme-responsive CRFP is best suited for foods with a well-defined, dominant spoilage enzyme signature and is unsuitable for frozen or ultra-high temperature (UHT) products where enzyme activity is negligible or for foods with complex, redundant enzyme profiles where selective triggering becomes unreliable. These considerations underpin the rational design of enzyme-responsive systems for precise, context-adaptive active agent release in food preservation.

### 3.3. Humidity-Response System

Moisture is a primary driver of food spoilage, contributing to microbial proliferation and accelerating the deterioration of product quality [38,39]. Elevated RH within food packages, often resulting from respiration, microbial metabolism, or environmental infiltration, creates conditions favorable for microbial growth [40]. Humidity-responsive packaging materials represent a key innovation in active food packaging, designed to detect internal moisture levels and trigger the release of functional agents like antimicrobials to inhibit spoilage and preserve food quality.

Polymers typically possess high cohesive energy due to strong intermolecular forces mediated by polar groups. Upon exposure to moisture, water interacts with the polymer matrix through three primary mechanisms: hydrogen bonding with polar groups, hydrogen bonding with pre-bound water molecules, and free-state dispersion interactions [41]. These interactions disrupt intermolecular forces, weaken cohesion, and enhance polymer plasticization [42]. According to the free-volume theory [43], increased molecular spacing due to water uptake accelerates the motion of polymer chains, facilitating the diffusion and release of small-molecule bioactives [44]. Such moisture-induced structural changes or specific chemical reactions thus enable controlled release of functional agents in humidity-responsive packaging systems.

#### 3.3.1. Physical Changes

**Figure 4:**
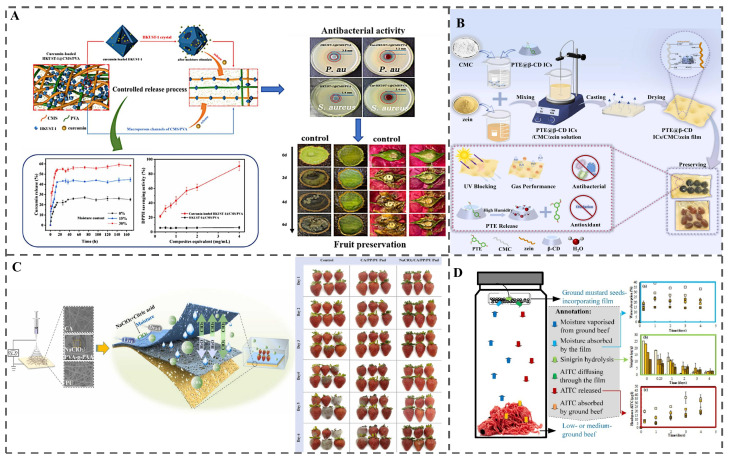
Humidity-responsive systems for food preservation. (**A**) Cur-HKUST-1@CMS/PVA film for fruit preservation [46]; (**B**) humidity-responsive mechanism of pterostilbene@β-cyclodextrin inclusion complexes/carboxymethyl cellulose/zein composite film [50]; (**C**) moisture-triggered ClO_2_ release and antibacterial mechanism of the triple-layer CA/PVA-g-PAA/PU nanofiber pad, with strawberry preservation photos [53]; (**D**) moisture-triggered AITC release from ground mustard seed film and interaction with ground beef [54].

Physical humidity responsiveness commonly involves exploiting moisture-induced alterations in polymer molecular spacing to release encapsulated bioactive compounds [45]. For instance, Liang et al. developed a multifunctional bio-based film (Cur-HKUST-1@CMS/PVA) by cross-linking carboxymethyl starch (CMS)/PVA with HKUST-1 MOF and incorporating curcumin (Cur) [46] (Figure 4A). The hydrophilic CMS component promotes water adsorption, leading to structural disruption of HKUST-1 and consequent release of Cur. Additionally, Luo et al. demonstrated that cinnamon essential oil (CEO) released from PEG-PCL micelles reached 72% after seven days at 75% RH [47]. This release behavior is attributed to water-PEG interactions, which accelerate the hydrolytic degradation of the PCL segment and consequently promote the diffusion of the encapsulated oil. Building on this, they fabricated a composite card combining 1-methylcyclopropene (1-MCP)/PVA and CEO-loaded micelles. During apricot storage, fruit transpiration and respiration increase the relative humidity inside the package. This elevated RH acts as an endogenous physiological stimulus. On one hand, water molecules interact with the hydrophilic PEG segment of the micelles, accelerating hydrolytic degradation of the hydrophobic PCL core, triggering on-demand release of encapsulated CEO. The released CEO exerts a strong antifungal effect against *Alternaria alternata*. On the other hand, the hydrophilic PVA film swells under high humidity, enabling sustained release of 1-MCP, which specifically blocks ethylene receptors and inhibits the ethylene-mediated ripening. This dual humidity-responsive synergistic system effectively reduced the decay index of apricots to only 11% after 8 days of storage at 25 °C, significantly lowered the peak respiration rate and ethylene production, delayed fruit softening and nutritional quality deterioration, and greatly extended the commercial shelf life of post-harvest apricots [48]. Cyclodextrin (CD), characterized by a conical structure with a hydrophobic cavity and hydrophilic exterior, form host-guest inclusion complexes [49,50]. Under high RH conditions, hydrogen bonds between guest molecules and the CD host weaken, disrupting the inclusion complex and triggering rapid release of the guest molecules from the CD cavity (Figure 4B) [51,52].

#### 3.3.2. Chemical Reactions

In addition to physical changes, moisture can also trigger chemical reactions with specific compounds to enable targeted antimicrobial release. Chlorine dioxide (ClO_2_), a broad-spectrum antimicrobial agent, is widely used in food preservation [55,56,57]. In humidity-responsive systems, ClO_2_ is generated in situ from the reaction between chlorite salts and food-grade acids, thereby facilitating controlled release. Du et al. engineered a three-layer nanofibrous pad that selectively absorbs excess moisture, initiating the reaction between sodium NaClO_2_ and citric acid to release ClO_2_ (Figure 4C) [53]. This system effectively suppressed microbial growth and extended the shelf life of strawberries. Allyl isothiocyanate (AITC), a volatile antimicrobial compound derived from mustard seeds, is produced via the enzymatic hydrolysis of sinigrin (a glycoside) by myrosinase—a process that is strongly moisture-dependent [58,59]. Bahmid et al. developed an antimicrobial cellulose acetate film incorporating finely ground mustard seeds [54]. Within this system, moisture vapor absorbed by the film activates myrosinase in the embedded mustard, triggering AITC formation within the polymer matrix. The rate and extent of AITC release are influenced by moisture uptake, and higher temperatures and RH further accelerate its diffusion into the surrounding environment (Figure 4D).

Although humidity-responsive packaging has demonstrated promising performance, its practical deployment remains highly condition-dependent. Unlike idealized laboratory settings, where systems are almost exclusively evaluated at fixed RH levels such as 75% or 95% RH, real food packages experience dynamic, non-uniform humidity fluctuations driven by respiration, condensation, and localized moisture pockets. This mismatch frequently causes premature activation or insufficient triggering, severely compromising on-demand release performance [46,50]. Another inherent drawback is that most moisture-induced structural changes are irreversible, resulting in a one-off burst release rather than sustained, controllable delivery. Once activated, these systems cannot be reinitialized or maintain long-term protection, making them unsuitable for foods requiring prolonged or multi-phase preservation [60].

From a scalability perspective, the feasibility of different humidity-responsive systems varies dramatically. Simple designs such as moisture-activated ClO_2_-releasing pads have already reached commercial readiness due to their low cost and reliance on GRAS-listed components [53]. In contrast, MOF-based humidity-responsive platforms rely on solvothermal synthesis, organic solvents, and complex post-modification, which pose substantial challenges for production costs, batch consistency, and regulatory approval for food-contact applications [46,61]. Despite their exceptional loading capacity and tunable release profiles, the technology readiness level of these advanced materials for commercial food packaging remains low.

The effectiveness of humidity-triggered activation is closely linked to the moisture evolution characteristics of the packaged food. humidity-responsive systems are most effective for high-respiration products such as strawberries, mushrooms, and fresh-cut produce, where rapid internal moisture accumulation provides reliable triggering signals. Conversely, this strategy is poorly suited for low-water-activity foods (e.g., nuts, biscuits, and dried commodities), where insufficient moisture fails to activate the system. It is also not ideal for products requiring weeks-long, low-dose sustained release, as irreversible one-shot activation cannot meet long-term preservation demands. Therefore, successful deployment demands precise matching between the moisture evolution profile of the target food and the activation threshold of the packaging material, with clear recognition that this technology is best suited for short-to-medium shelf-life foods with predictable and rapid humidity development.

### 3.4. Temperature-Response System

Temperature variations during food storage and transportation critically affect food quality by regulating microbial growth, enzymatic activity, and chemical reaction rates [2,62]. Elevated temperatures promote processes such as lipid oxidation, protein degradation, microbial proliferation, and ethylene release in climacteric fruits, collectively accelerating food deterioration [63]. To counteract these effects, temperature-responsive packaging systems are designed to dynamically adjust the internal environment, thereby extending shelf life and ensuring food safety.

**Figure 5:**
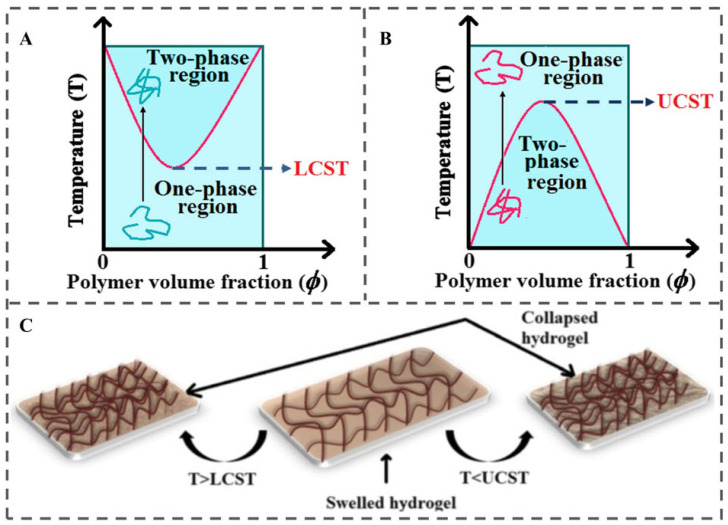
Schematic representation of phase transitions in polymer solutions and hydrogels: (**A**) LCST behavior, (**B**) UCST behavior, and (**C**) volume transitions in hydrogels above LCST and below UCST [65].

The fundamental principle of temperature-responsive packaging lies in exploiting the reversible physicochemical transitions of thermosensitive polymers in response to temperature fluctuations. These transitions—such as changes in solubility, hydrophilic-hydrophobic balance, and structural conformation—are harnessed to trigger controlled release of encapsulated bioactive compounds [7,64]. Among the diverse thermoresponsive behaviors observed in these materials, two primary mechanisms predominate: the Low Critical Solution Temperature (LCST) behavior and the Upper Critical Solution Temperature (UCST) behavior (Figure 5).

#### 3.4.1. LCST-Based Response Mechanism

The LCST is defined as the temperature at which a polymer transitions from a soluble to an insoluble state in an aqueous solvent, often accompanied by phase separation [66]. Below the LCST, polymers such as poly(N-isopropylacrylamide) (PNIPAAm) exhibit hydrophilic properties and remain soluble due to intrachain hydrogen bonding. Above the LCST, however, the polymer undergoes a hydrophilic-to-hydrophobic transition, accompanied by dehydration, chain collapse, and a reduction in hydrophilicity [7]. This transformation can facilitate the release of encapsulated bioactives. For instance, Xia et al. developed a temperature-responsive nanofiber membrane (PLP) for blackberry preservation via electrospinning, incorporating PLA fibers loaded with lemon essential oil (LEO) and coated with PNIPAAm [67]. At temperatures below the LCST of PNIPAAm, the hydrophilic extended PNIPAAm layer acts as a physical barrier to inhibit the premature volatilization and release of LEO encapsulated in the PLA fiber core. In contrast, at temperatures above the LCST, PNIPAAm underwent a hydrophobic transition and collapsed into a globular structure, facilitating LEO release. This temperature-controlled release behavior endowed the PLP membrane with long-lasting antibacterial and antioxidant activities, effectively inhibited the decay and softening of blackberries under fluctuating temperature conditions, maintained the nutritional quality of the fruit, and significantly extended the shelf life of post-harvest blackberries.

#### 3.4.2. UCST-Based Response Mechanism

Polymers exhibiting UCST behavior display the opposite transition: they are insoluble or poorly soluble below the UCST but become highly soluble above it [6]. A classic example is the interpenetrating network (IPN) hydrogel composed of polyacrylamide (PAAm) and PAA, wherein hydrogen bonding interactions between the polymer chains lead to temperature-dependent solubility changes. Poly(N,N-Dimethylacrylamide) (PDMAAm) (a hydrogen-bond acceptor) and PAA (a hydrogen-bond donor) form complexes that dissociate above the UCST, resulting in increased solubility and hydrogel swelling. For instance, Aoki et al. demonstrated that PDMAAm/PAA IPNs exhibited 0% transmittance at low temperatures and 100% transmittance above the UCST, confirming the dissociation of intermolecular complexes [68]. This UCST-driven swelling enables pulsatile release of entrapped bioactives, making it suitable for high-temperature-triggered delivery systems. Similarly, Wang et al. fabricated a UCST-responsive IPN hydrogel from PAAm and β-cyclodextrin-grafted PAA (PAA-graft-β-CD), with a UCST of 35 °C [69]. Swelling occurred above this temperature, promoting the release of encapsulated bioactives.

In addition, the transition temperature of thermosensitive polymers can be modulated by the addition of additives, such as surfactants, salts, or co-solvents, which alter the polymer-solvent interactions or modify the hydrophilicity–hydrophobicity balance. Such adjustments provide a means to fine-tune the thermoresponsive characteristics of packaging materials for specific applications [64].

Most existing studies validate temperature-responsive systems under steady-state conditions. In reality, however, cold chains are characterized by repeated heating-cooling cycles and dynamic fluctuations arising from door openings, transport interruptions, or equipment failures. Under such fluctuating conditions, LCST- and UCST-type polymers exhibit pronounced thermal hysteresis: the phase transition during cooling does not mirror that during heating. When the temperature rises above the transition point, polymer chains collapse from an extended, hydrated state into compact, dehydrated globules, triggering the release of encapsulated bioactives. Upon subsequent cooling, however, these collapsed globules do not instantaneously re-expand at the same temperature; instead, they remain trapped in the collapsed state due to hydrophobic chain-chain associations and slow water regeneration. As a result, the system requires cooling to a significantly lower temperature to revert to its original hydrated conformation. This thermal hysteresis leads to quasi-irreversible behavior under dynamic cold-chain conditions, where repeated temperature fluctuations can cause incomplete release, diminished responsiveness, or failure to shut off release effectively [70]. Moreover, the majority of synthetic temperature-responsive polymers, such as PNIPAAm, lack established food-contact authorization. Hence, despite their excellent laboratory performance, most synthetic thermosensitive polymers remain commercially non-viable for food packaging.

The practical value of temperature-responsive CRFP ultimately depends on the temperature sensitivity and storage profile of the target food. Such systems are most applicable to products requiring strict cold-chain integrity, such as fresh meat, frozen seafood, and ice cream. In contrast, they are less effective for shelf-stable products (e.g., canned goods, dry foods, ambient dairy) or for foods relatively insensitive to moderate temperature fluctuations. Successful implementation therefore requires careful alignment of the polymer’s transition temperature with the temperature sensitivity and storage requirements of the target food.

### 3.5. Light-Response System

**Figure 6:**
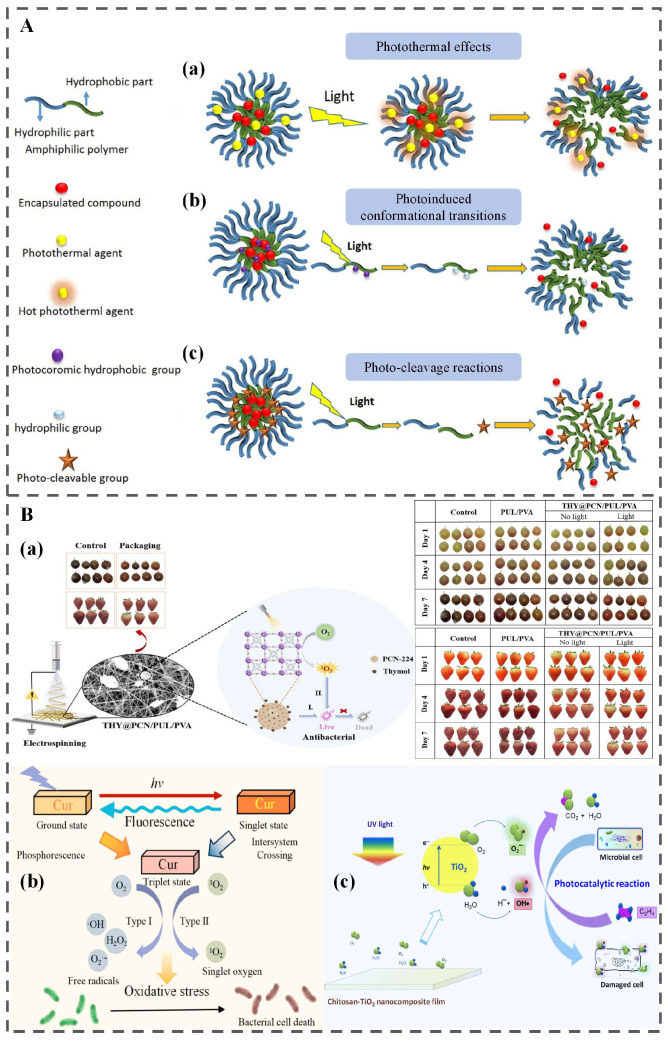
Light-responsive release mechanisms in food applications. (**A**) Mechanisms of light-triggered release of encapsulated bioactives from carriers [6]; (**B**) photogenerated ROS production: (**a**) ROS production of THY@PCN/PUL/PVA nanofibers, with grapes and strawberries preservation effects [73]; (**b**) ROS production of KC/Cur-OSAS (K-carrageenan with curcumin and octenyl succinic anhydride starch) [74]; (**c**) mechanism of photocatalytic degradation of ethylene and antimicrobial activity of the chitosan-TiO_2_ nanocomposite film [75].

Apart from thermally induced transitions, external physical stimuli such as light irradiation offer an alternative, non-contact triggering strategy [71]. Light energy within the ultraviolet (UV) and visible (Vis) regions plays a pivotal role in food quality deterioration by triggering degradation and oxidation reactions [72]. These photoinduced processes subsequently lead to multiple adverse effects, including nutrient depletion, loss of bioactive compounds, the development of off-odors and off-flavors, color fading, and the formation of potentially harmful substances. This approach offers distinct advantages over other external stimuli-responsive systems, particularly its precise control over intensity, wavelength, and exposure duration [6]. Current research on light-responsive release systems in food applications primarily focuses on four mechanisms: (1) photoinduced conformational transitions; (2) photo-cleavage reactions; (3) photothermal effects; and (4) photogenerated reactive oxygen species (ROS) production, as shown in Figure 6.

#### 3.5.1. Photoinduced Conformational Transitions

Azobenzene serves as a paradigmatic light-responsive molecule, characterized by its -N=N- azo bond, which undergoes a geometric transformation upon irradiation with UV light, triggering a well-documented conformational transition from the thermodynamically stable trans-isomer to the metastable cis-isomer. Critically, this isomerization is fully reversible: exposure to visible light or mild thermal stimuli promotes the complete reversion of the cis-isomer back to the trans-isomer [76,77,78]. Marturano and colleagues developed a light-responsive polymer nanocapsule (NC) system, embedding azobenzene units within the capsule shell to mediate photo-triggered functionality [79]. Upon UV irradiation, the trans-to-cis isomerization of azobenzene initiated, driving conformational rearrangement of the NC shell and enabling controlled release of encapsulated essential oils (EOs) and the model drug coumarin-6. Building on this foundation, the same research group subsequently extended the application of this system to antimicrobial active packaging [80]. They incorporated thyme EO-loaded photo-responsive NCs as functional coatings onto commercial polyethylene (PE) and PLA films. 24 h after a 15 min UV exposure, the concentration of thyme EO in the headspace of the coated films was eightfold higher than that of unirradiated counterparts, validating the efficiency of the light-triggered release mechanism for on-demand antimicrobial delivery.

#### 3.5.2. Photo-Cleavage Reactions

In this mechanism, photo-cleavable moieties—including O-nitrobenzyl (ONB), coumarin, and 4-bromo-7-hydroxycoumarin (BHC)—are integrated into the polymer backbone of the delivery system. Upon targeted light irradiation, these photo-sensitive groups undergo a photochemical cleavage, leading to polymer backbone degradation and the on-demand release of encapsulated bioactive compounds relevant to food preservation [81]. Fomina and collaborators designed a novel light-responsive polymer with multiple photo-sensitive units along its backbone, incorporating a quinone-methide (QM) self-immolative moiety [82]. This polymer was fabricated into nanoparticles (NPs) for encapsulating Nile Red, serving as a model hydrophobic functional molecule analogous to bioactives such as hydrophobic antimicrobials or natural antioxidants. Upon specific light irradiation, two sequential processes were observed: first, a triggered burst release of the encapsulated payload (Nile Red), followed by progressive degradation of the NP matrix. Importantly, this system demonstrates high versatility, as its photo-sensitive triggering groups can be tailored to respond to a broad range of light wavelengths, offering adaptability to diverse food packaging scenarios.

#### 3.5.3. Photothermal Effects

Photothermal nanomaterials, exemplified by Au NPs, are widely investigated for food-related applications due to their ability to absorb near-infrared (NIR) light and convert it into thermal energy [83,84]. When integrated into bioactive delivery carriers for food preservation, the locally generated heat upon NIR irradiation induces structural disruption of the carrier matrix, thereby enabling on-demand release of encapsulated bioactive compounds. Chiang et al. developed a photothermally responsive injectable system based on hollow microspheres (HMs), featuring a PLGA shell encapsulating an aqueous core containing vancomycin (Van, an antibiotic targeting MRSA) and polypyrrole nanoparticles (PPy NPs, serving as photothermal agents) [85]. Upon NIR irradiation, the localized temperature exceeds PLGA’s glass transition temperature, causing the shell to transition from a glassy to a rubbery state. This structural change facilitates the rapid release of Van. Notably, this NIR-triggered photothermal mechanism holds significant potential for food packaging. By substituting Van with food-grade bioactives (e.g., curcumin, antimicrobial peptides), the HM system can be adapted for use in active packaging or edible coatings. Under NIR irradiation, it still induces PLGA shell phase transition, enabling controlled release of bioactives and effectively inhibiting microbial spoilage and oxidation while meeting food safety standards.

#### 3.5.4. Photogenerated ROS Production

ROS exhibit dual functionality in food preservation: on one hand, they can interact with polyunsaturated phospholipids in the cell membranes of spoilage microorganisms, thereby disrupting membrane integrity and inactivating microbes (Figure 6B) [75]; on the other hand, they can oxidize ethylene to non-toxic end products (CO_2_ and H_2_O) [86,87,88]. Collectively, these properties highlight ROS’s antimicrobial activity and ethylene-scavenging capacity, making it a promising functional component for extending the shelf life of fresh foods. Various ROS-generating NPs have been developed for antimicrobial food packaging, including berberine-based NPs [89,90,91], titanium dioxide (TiO_2_) NPs [92,93,94], PCN-224 (a zirconium-based porphyrin MOF) [73], and Cur-loaded NPs [74,95].

Light-responsive systems offer the unique advantage of non-contact, spatiotemporally controlled release, yet a persistent challenge lies in the potential conflict between the activation mechanism and the primary goal of food preservation. Light, particularly UV and high-energy visible wavelengths, is a well-established contributor to food quality degradation, including lipid oxidation, vitamin loss, pigment fading, and off-flavor development. Therefore, using light as a trigger for active compound release, under certain scenarios, introduces a trade-off: while the system may deliver antimicrobials or antioxidants upon targeted irradiation, undesired light exposure might simultaneously accelerate spoilage in photosensitive products such as dairy, oils, and certain fruits and vegetables. However, it should be noted that photo-responsive packaging typically employs specific, controllable light sources for activation, which can be managed to minimize adverse effects on food quality.

A second, often underestimated barrier concerns the safety and regulatory fate of light-responsive components. Photosensitizers, whether synthetic, nanomaterial-based, or even naturally derived, may migrate into the food matrix, and their photodegradation byproducts remain rarely characterized. This knowledge gap poses significant hurdles for regulatory approval in food-contact applications.

From a pragmatic standpoint, light-responsive CRFP is best suited for transparently packaged products where the food surface is directly accessible to illumination, for instance, fresh-cut produce, ready-to-eat salads, or deli meats in clear overwraps. It is not a good fit for opaque or multi-layer packaging, light-sensitive commodities or foods stored in dark supply chains. Consequently, rather than a broadly applicable solution, light-responsive systems are likely to remain niche technologies for high-value, transparently packaged applications unless the intertwined challenges of light-food compatibility, photosensitizer safety, and regulatory acceptance are systematically resolved.

### 3.6. Other Stimuli

Beyond single-stimulus systems, emerging responsive modalities are being explored to enhance the adaptability of food packaging. For instance, Yu et al. developed a glutathione-responsive polymer (Cos-G3-Dns) that releases SO_2_ upon activation, inhibiting foodborne pathogens through ROS overproduction and thiol depletion [96]. Huang et al. fabricated a phosphate-responsive antibacterial carrier (HKUST-1@CMCS) by hybridizing carboxymethyl chitosan with HKUST-1 for controlled release of dimethyl fumarate [97].

However, single-stimulus systems often lack adaptability to the complex, multi-stimuli environments typical of food storage [2,62]. To address this, multi-stimulus-responsive systems integrating two or more triggers are gaining attention for more precise, sequential, or synergistic control. Examples include pH/amylase dual-responsive THY@PLA-COS-DAS (THY-loaded PLA nanofibers modified with chitosan oligosaccharide and dialdehyde starch) membranes for melon preservation [98] and pH/humidity-responsive LA@Cu-MOF/SA films for fruit preservation [99]. Table 1 summarizes representative corrective responsive systems.

Despite significant progress in stimulus-responsive packaging, real-world implementation remains hindered by a wide gap between idealized lab conditions and complex food supply chain dynamics. Most studies optimize performance under single-stimulus, static environments, but actual spoilage involves dynamic, multi-factorial fluctuations. This discrepancy severely undermines system robustness: pH-responsive materials often fail to discriminate subtle spoilage-related shifts from background variations; enzyme-responsive carriers struggle with non-specific cross-reactivity in complex microbiota; and humidity, temperature, and light systems are frequently compromised by matrix-specific interferences such as lipid interactions or protein adsorption. Furthermore, multi-stimulus integration remains in its infancy, with limited understanding of signal modulation under real spoilage conditions. These limitations underscore the need for mechanism-driven research focused on operational boundaries, matrix compatibility, and cross-stimulus effects, rather than just demonstrating on-demand release. Such critical insights are essential to guide targeted design and unlock practical potential.

## 4. Key Materials and Carrier Technologies

The previous section systematically introduced various stimulus-responsive mechanisms. However, these mechanisms cannot function in isolation, and they require suitable materials and carrier structures. The translation of these conceptual triggering principles into practical, efficient, and scalable food preservation strategies depends fundamentally on the rational design, selection, and integration of high-performance functional materials and advanced encapsulation systems. Specifically, the capacity of a responsive system to perceive environmental stimuli and subsequently implement controlled release of bioactives is fundamentally governed by the intrinsic properties of its constituent materials. Encapsulation systems, meanwhile, serve as the structural bridge between responsive materials and bioactive payloads, not only protecting labile compounds from premature degradation but also precisely regulating release kinetics to align with the dynamic changes in the food microenvironment. Accordingly, this section focuses on the core materials and carrier technologies that enable the aforementioned stimulus-responsive mechanisms, elucidating their structural characteristics, functional advantages, and synergistic contributions to the advancement of CRFP.

### 4.1. Materials

#### 4.1.1. Mesoporous Silica Nanoparticles (MSNs)

**Figure 7:**
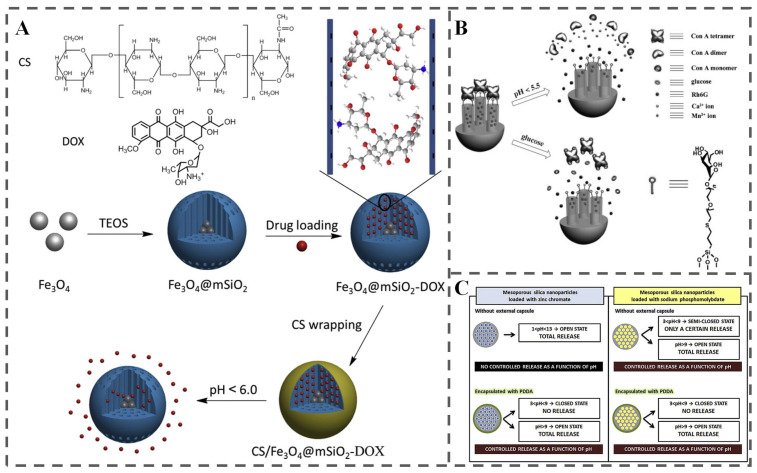
Examples of stimuli-responsive MSN-based carrier systems. (**A**) pH-sensitive controlled release from mesoporous magnetic nanocomposites with chitosan gatekeepers [105]; (**B**) controlled release of cargo from Con A-gated MSN nanocontainers responsive to pH and glucose [108]; (**C**) pH-responsive mesoporous silica release [104].

MSNs are nanoscale materials characterized by their uniform pore structure and large internal surface area. These unique properties, combined with attributes like high loading capacity, good chemical stability, inherent biocompatibility, tunable pore size, and facile surface functionalization, have positioned MSNs as versatile platforms for a wide range of applications, including drug delivery, catalysis, and biosensing [102,103,104]. In the context of CRFP, MSNs are primarily employed as efficient carriers for encapsulating and delivering functional components directly into the food matrix or onto packaging surfaces. The inherent stimuli-responsiveness of MSNs can be further enhanced through surface modification. For instance, coating MSN surfaces with stimuli-responsive polymers like chitosan or cyclodextrin imparts responsiveness to environmental triggers such as pH or humidity (Figure 7A) [105,106,107].

Several studies have demonstrated the potential of MSNs for targeted and controlled release in food systems. One example involves mannose-functionalized MSNs gated by Concanavalin A (Con A), developed by Wu et al. (Figure 7B) [108]. Here, MSNs were functionalized with mannose ligands, and their pores were sealed using Con A via multivalent carbohydrate-protein interactions, with Rhodamine 6G serving as a model cargo. This system exhibited dual responsiveness: acidic conditions triggered cargo release by disrupting the Con A quaternary structure and facilitating the dissociation of Ca^2+^/Mn^2+^ ions that help maintain the pore structure. High glucose concentrations also induced release, likely by competing for the Con A binding sites, thereby displacing Con A from the mannose ligands. Another study utilized MSNs loaded with sodium phosphomolybdate, an environmentally friendly corrosion inhibitor (Figure 7C) [104]. The findings revealed pH-dependent release kinetics: unencapsulated sodium phosphomolybdate within MSNs showed controlled release inhibited within the pH range of 3–9 due to polymerization of molybdenum species and pH-dependent changes in phosphate speciation. Release was significantly enhanced above pH 9, and complete release occurred at pH 13.

These investigations highlight the potential of MSNs for stimuli-responsive release in food-related applications. While current research on stimuli-responsive MSNs has predominantly concentrated on pharmaceutical drug delivery and chemical applications [109,110,111], the underlying principles and material characteristics offer valuable insights and a promising foundation for developing the next generation of corrective food-responsive packaging systems.

#### 4.1.2. Metal Oxide

Metal oxides represent a class of materials gaining significant attention for their inherent stimuli-responsive properties in responsive food packaging. Notable examples include copper oxides, iron oxide, and particularly zinc oxide (ZnO) and TiO_2_, which are the most widely investigated and utilized metal oxides for antimicrobial applications [112,113,114].

**Figure 8:**
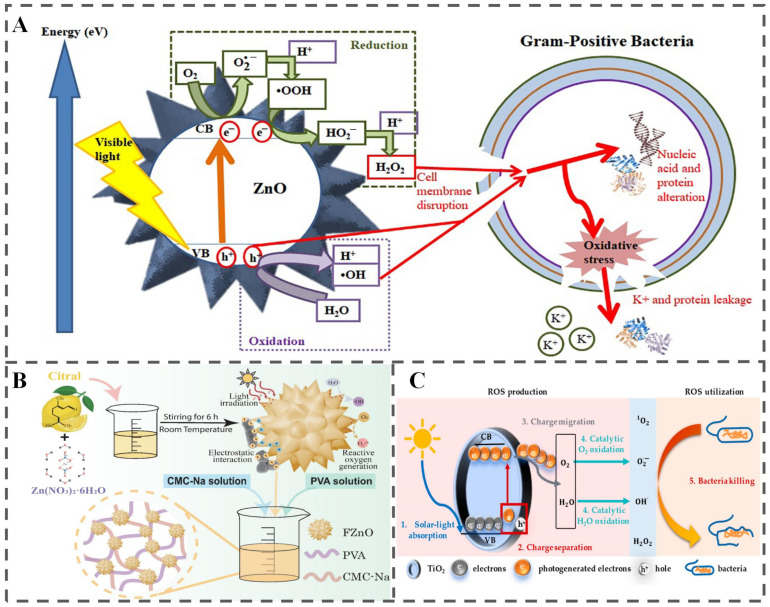
Stimuli-responsive metal oxide antibacterial systems. (**A**) Flower NPs, shown to generate various ROS upon visible light illumination [115]; (**B**) ROS production of citral-modified, flower-like ZnO NPs [120]; (**C**) Photocatalytic and antibacterial mechanisms of nano-TiO_2_ [121].

ZnO, approved by the U.S. Food and Drug Administration (FDA) as GRAS, is especially valued for its efficacy as an antibacterial and antifungal agent, making it a cornerstone in active food packaging development [113]. Leveraging their photocatalytic nature, ZnO nanoparticles generate ROS on their surface upon exposure to UV light, thereby exhibiting enhanced bioactivity (Figure 8A) [115]. Capitalizing on this, numerous studies have developed ZnO nanoparticle-incorporated packaging films [116,117,118,119]. For instance, Li et al. synthesized citral-modified, flower-like ZnO nanoparticles using an organic-inorganic hybrid strategy (Figure 8B) [120]. The resulting film, consisting of flower-like ZnO embedded in a polyvinyl alcohol/sodium carboxymethyl cellulose matrix, effectively prolonged the shelf life of cherry tomatoes.

Similarly, nano-TiO_2_ is renowned for its potent photocatalytic and antibacterial properties, which are also attributed to ROS generation (Figure 8C) [121]. Xu et al. developed a visible light-activated antibacterial film by embedding tetra(4-carboxyphenyl)porphyrin (TcPP)-sensitized TiO_2_ nanoparticles into cellulose nanofiber (CNF) films [122]. The TcPP sensitizer absorbed visible light and injected electrons into the TiO_2_ conduction band, effectively generating ROS even under visible light illumination. The antibacterial efficacy of this film was shown to correlate directly with light intensity. However, despite their promising antimicrobial effects, concerns regarding the biocompatibility and potential safety of TiO_2_ nanoparticles have emerged from recent safety evaluations [123]. These findings underscore the necessity for further research and rigorous assessment before the large-scale, commercial application of TiO_2_ in food packaging can be confidently pursued.

#### 4.1.3. MOFs

**Figure 9:**
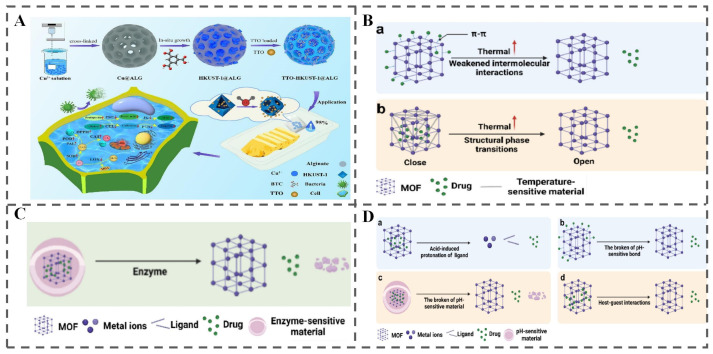
Stimuli-responsive MOFs for responsive food packaging: examples and mechanisms. (**A**) MOF-based moisture-responsive essential oil hydrogel bead (TTO-HKUST-1@ALG) and its function [60]; mechanisms of MOF-based (**B**) temperature-response, (**C**) enzyme-response and (**D**) pH-response [13].

MOFs, constructed from metal nodes coordinated with organic linkers, are crystalline porous materials distinguished by their exceptionally large specific surface areas, highly tunable pore structures, and facile surface modification capabilities. These inherent properties make them a highly promising carrier system for active ingredients in various applications [13,124]. Among various MOFs, Cu-based HKUST-1 has gained significant attention for food preservation applications. For example, Tian et al. developed hydrogel beads (TTO-HKUST-1@ALG, Figure 9A) by combining alginate-copper ion cross-linking, in situ MOF growth, and self-assembly techniques to load tea tree essential oil (TTO) for RH-responsive fresh-cut pineapple preservation [60]. As the RH increased from 45% to 95%, the HKUST-1 structure underwent rapid disassembly, leading to a corresponding increase in TTO release from 33.89% to 70.98%. Furthermore, the stimuli-responsiveness of MOFs extends beyond humidity. These materials can be engineered to respond to various other triggers, including pH changes, temperature fluctuations, and biological stimuli [13]. The underlying mechanisms for these diverse responses, as illustrated in Figure 9B–D, highlight the versatility of MOFs as stimuli-responsive carriers for targeted delivery in responsive food packaging applications.

#### 4.1.4. Polymers

**Figure 10:**
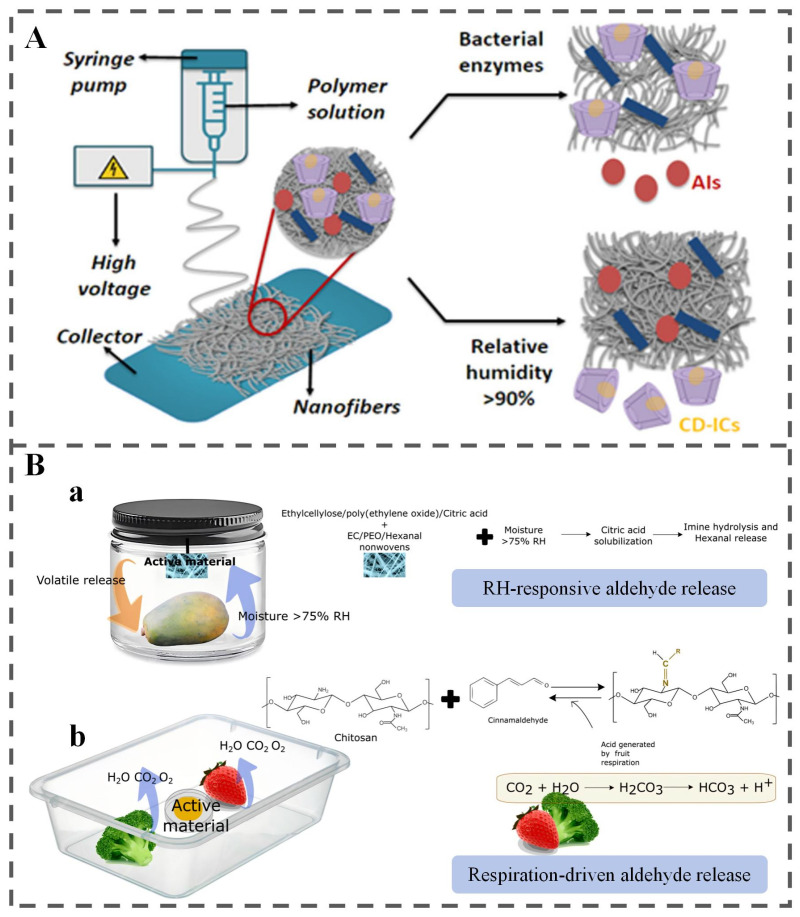
Stimuli-responsive polymers for responsive food packaging. (**A**) Representation of the enzyme- and RH-triggered strategies [101]; (**B**) reversible covalent bond-based responsive polymer designs [128].

Stimuli-responsive polymers have gained increasing attention in active food packaging owing to their ability to enhance food safety, extend shelf life, and enable controlled release of functional agents [125,126,127]. For instance, enzyme- and humidity-responsive antimicrobial fibers fabricated from cellulose nanocrystals (CNCs), zein, and starch exhibit targeted degradation under microbial enzyme action, thereby triggering thymol release, while cyclodextrin inclusion complexes facilitate humidity-regulated antimicrobial delivery [101] (Figure 10A). In addition, Esteve-Redondo et al. reviewed strategies for the controlled delivery of antimicrobial volatiles via reversible covalent bonding, highlighting the importance of designing materials responsive to environmental cues [128]. For example, moisture-responsive hexanal release has been achieved using bilayer electrospun ethylcellulose (EC)/poly(ethylene oxide) (PEO) systems, in which an acid-labile imidazolidine precursor undergoes hydrolysis triggered by humidity-induced citric acid diffusion [129] (Figure 10B(a)). Moreover, nanocellulose-based materials have likewise emerged as versatile platforms for incorporating bioactive compounds due to their tunable surface chemistry and biocompatibility [130,131]. These systems can be rationally engineered to respond to specific stimuli, enabling controlled release in active packaging applications. Overall, recent advances underscore the significant potential of stimuli-responsive polymers to enhance food safety and sustainability through precise, environment-triggered release mechanisms [101,128,130].

Despite substantial progress in the development of advanced responsive materials, their translational readiness varies considerably. Conventional polymer matrices such as chitosan, starch derivatives, and synthetic food-contact polymers benefit from relatively well-established regulatory pathways under frameworks such as EU Regulation No. 10/2011 on plastic materials intended for food contact. These materials often incorporate components with existing food-contact authorization or GRAS status, thereby facilitating industrial scalability and regulatory approval.

In contrast, highly engineered materials, including MOFs, metal oxide nanoparticles, and photoresponsive nanocomposites, remain largely confined to laboratory-scale studies. Although MOFs offer exceptional loading capacity, structural tunability, and stimulus responsiveness, recent critical reviews have highlighted unresolved concerns regarding framework stability, potential leaching of metal ions, and structural degradation under acidic or high-moisture food environments, all of which may influence migration behavior and long-term safety [61,123,132]. Similarly, the safety of nano-sized TiO_2_ has been re-evaluated by the European Food Safety Authority (EFSA), which concluded that genotoxicity concerns could not be excluded, underscoring the regulatory uncertainty associated with metal oxide-based systems [133]. Moreover, EFSA’s guidance on risk assessment of nanomaterials in the food and feed chain [134] emphasizes the need for extended toxicological evaluation and migration testing, significantly increasing approval timelines and development costs.

Therefore, despite promising performance at the laboratory level, these nanostructured systems encounter greater regulatory and translational hurdles compared with polymer-based materials that incorporate authorized food-contact constituents. To better contextualize the practical relevance of the aforementioned materials, a comparative assessment of their technological maturity and translational feasibility is summarized in Table 2.

### 4.2. Carrier Technologies

Although responsive materials determine stimulus sensitivity, effective preservation depends on suitable carrier architectures to encapsulate and regulate bioactive release. Carrier technologies thus bridge material responsiveness and practical application performance. The following section highlights representative carrier systems enabling stimulus-responsive strategies.

#### 4.2.1. Micelles

Micelles, as nanocarrier systems formed by the self-assembly of amphiphilic molecules possessing both hydrophilic and hydrophobic moieties, represent a promising class of materials for stimuli-responsive food packaging. Their hydrophobic core effectively encapsulates lipophilic active compounds [135]. This encapsulation offers dual benefits: it protects active ingredients from detrimental environmental factors and enhances their dispersibility and stability within aqueous food matrices. Furthermore, micelles can be designed to load and deliver a variety of bioactive agents, releasing them in response to specific environmental triggers. This functionality enables precise control over the release of active ingredients, thereby extending food shelf life and providing robust protection for labile compounds, while offering excellent adaptability for advanced responsive packaging applications [62,136]. While humidity-responsive micelles have been previously discussed in the context of specific response mechanisms [47,48], the literature also encompasses a wider range of stimuli-responsive micelle systems.

**Figure 11:**
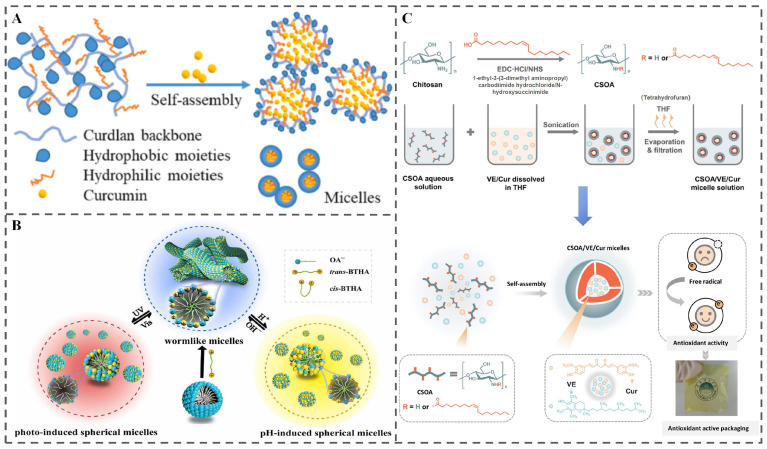
Stimuli-responsive micelles for responsive food packaging. (**A**) Formation of OSA-CMCD micelles [137]; (**B**) photo/pH dual-responsive wormlike micellar system [138]; (**C**) vitamin E and Cur co-loaded chitosan-graft-oleic acid (CSOA) micelles as potential antioxidant additives [135].

For instance, Li et al. synthesized amphiphilic octenyl succinic anhydride-modified carboxymethyl curdlan (OSA-CMCD) polymers with varying degrees of substitution for pH-responsive curcumin delivery, as illustrated in Figure 11A [137]. Similarly, Bi et al. constructed a photo/pH dual-responsive wormlike micellar system using the cationic surfactant 4,4′-bis(trimethylammonium-6-hexyloxy)azobenzene bromide (BTHA) and the anionic surfactant sodium oleate (NaOA) [138]. UV irradiation induced a trans-to-cis isomerization in BTHA, disrupting molecular packing at the micellar interface and causing wormlike micelles to transform into spherical structures—a transformation reversible under visible light. Modulating the pH influenced NaOA’s protonation state, enabling reversible changes in micellar morphology and solution viscosity, exemplifying materials with dynamic adaptability suitable for food packaging applications (Figure 11B). These examples highlight the potential of micelles for controlled release.

Future research endeavors will likely focus on developing multifunctional micellar systems, potentially combining capabilities such as antibacterial, antioxidant, and intelligent indicator functionalities. This can be achieved either by co-encapsulating diverse active agents within a single micelle or by designing complex, composite micellar architectures (Figure 11C) [135]. Additionally, integrating micelles with other nanomaterials holds promise for enhancing the mechanical strength and barrier properties of packaging materials while enabling more sophisticated controlled release strategies. Driven by increasing consumer demands for eco-friendliness and food safety, a significant research trend involves prioritizing natural, biodegradable, and food-grade micelle-forming materials. Utilizing biopolymers like chitosan, alginate, or food proteins such as casein and soy protein as building blocks for micelles will further enhance the safety profile of these systems for direct application in food packaging [139,140].

#### 4.2.2. Hydrogels

Hydrogels are defined as three-dimensional polymeric networks formed by cross-linking hydrophilic polymer chains, typically resulting from interactions between solid polymer components and water [141]. They possess exceptional water absorption/swelling capacity and porous structures, creating pathways for the permeation and adsorption of target molecules within the hydrogel matrix [142]. Crucially, upon exposure to specific external stimuli, many hydrogels demonstrate enhanced responsiveness towards incorporated substances, improving their selectivity [12].

**Figure 12:**
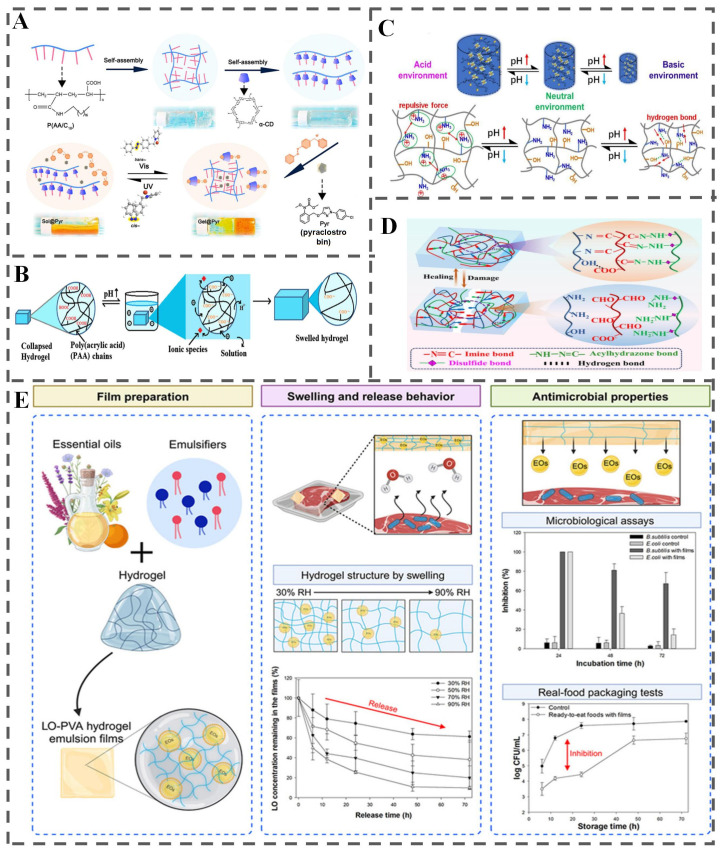
Stimuli-responsive hydrogels for responsive food packaging. (**A**) Sol–gel phase transition of C18-modified poly (acrylic acid) [77]; (**B**) controllable swelling behavior [65]; (**C**) dynamic conformational rearrangements [146]; (**D**) reversible dissociation-reorganization of dynamic covalent bonding and non-covalent interactions [147]; (**E**) mechanisms of LO-PVA hydrogel emulsion film preparation, humidity-responsive swelling/LO release behavior, and antimicrobial properties [145].

Responsive hydrogels exhibit remarkable potential for responsive packaging applications due to their ability to undergo reversible, rapid, and significant alterations in physicochemical properties when triggered by specific environmental cues [143]. This stimulus-responsiveness enables them to recognize and react dynamically to environmental changes, primarily through four key mechanisms (Figure 12A–D): (1) sol–gel phase transitions; (2) controllable swelling/deswelling behavior; (3) conformational rearrangements of the polymer network; and (4) reversible dissociation and reorganization of dynamic covalent and non-covalent interactions [144]. By rational designing the hydrogel composition and structure, targeted release of active ingredients can be achieved under specific conditions, thereby extending food shelf life or enhancing product quality.

A practical example is the humidity-responsive lemongrass essential oil (LO)-PVA hydrogel emulsion film developed by Ahn et al. [145]. As shown in Figure 12E, this system utilizes the hydrogel’s swelling property: increased RH causes PVA to absorb more water and swell, increasing gas permeability and consequently accelerating LO release. Despite their immense potential, relatively limited research has explored the application of stimulus-responsive hydrogels specifically within diverse food systems. This represents a significant opportunity for further investigation. Integrating these materials with key responsive components discussed in Section 4.1 offers a promising avenue for future work. Moreover, a critical need exists to identify and validate food-grade hydrogel precursors and formulations capable of exhibiting reliable, controlled responses to specific food-relevant stimuli [12].

#### 4.2.3. Lipid-Based Nanocarriers

**Figure 13:**
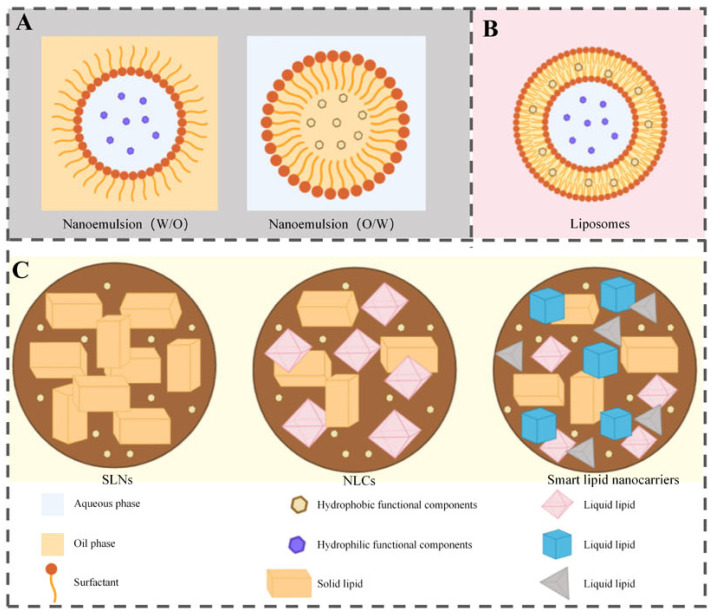
Lipid-based nanocarriers: (**A**) nanoemulsions; (**B**) liposomes; (**C**) various nanolipid carriers.

Lipid-based nanocarriers represent a significant and versatile class of nanoencapsulation systems for delivering functional ingredients across diverse food applications. This category encompasses three primary subgroups: nanoemulsions, nanoliposomes (also referred to as nanostructured phospholipid carriers), and nanolipid carriers (Figure 13A). Nanoemulsions include subtypes such as single, double, Pickering, and structural emulsions stabilized by emulsifiers or biopolymers [148,149]. Nanoliposomes, consisting of phospholipid bilayers arranged in unilamellar or multilamellar structures, are widely used to protect and deliver bioactive compounds in pharmaceutical, cosmetic, and food sectors [150,151,152]. Nanolipid carriers include solid lipid nanoparticles (SLNs), nanostructured lipid carriers (NLCs), and smart lipid nanocarriers [14,153]. SLNs feature a solid lipid matrix for bioactive entrapment [154], while NLCs combine solid and liquid lipids to improve loading efficiency [155]. Smart NLCs incorporate multiple solid lipids to form highly disordered matrices, enabling enhanced encapsulation and targeted delivery [14].

Beyond conventional encapsulation, increasing attention has been directed toward stimulus-responsive lipid carriers for controlled release in complex food microenvironments. For example, Jash and Rizvi developed pH-sensitive liposomes from milk fat globule membrane phospholipids using green supercritical-CO_2_ technology. Coating these liposomes with Eudragit^®^S100, a pH-responsive polymer, enabled gastric protection and targeted intestinal release [156]. Similarly, Lin et al. designed bio-responsive composite liposomes (FLPs) incorporating Litsea cubeba essential oil [157]. These liposomes featured an outer coating of L-fucose and an embedded silk fibroin layer. The bacterial enzyme HtrA, secreted by *C. jejuni*, hydrolyzes the silk fibroin, triggering the release of the encapsulated essential oil—a mechanism potentially exploitable for targeted antimicrobial delivery.

Rather than treating stimuli, carrier materials, and release strategies as independent design variables, CRFP systems should be developed through an integrated and application-driven framework. In practical food systems, preservation performance is governed not only by the presence of a trigger but also by the alignment between spoilage pathways and material response behavior. Therefore, effective CRFP design requires coordinated consideration of four interrelated dimensions: (1) the dominant spoilage mechanisms of the target food matrix; (2) controllable and reversible response mechanisms capable of operating within realistic fluctuation ranges; (3) detectable internal or external stimuli that reliably reflect quality deterioration; and (4) compatible carrier architectures that ensure stability, protection of bioactives, and precise release kinetics. Only when these elements are rationally matched can responsive systems achieve functional relevance under real storage conditions. To bridge the mechanistic principles discussed in Section 3 with the material and carrier platforms summarized in Section 4, a design-oriented framework is proposed to systematically guide CRFP development (Table 3).

## 5. Challenges and Future Perspectives

The integration of advanced materials with encapsulation technologies has enabled CRFP systems capable of on-demand bioactive release in response to food microenvironmental cues, marking a paradigm shift beyond conventional passive preservation strategies [36,136,158]. However, several critical bottlenecks hinder their widespread adoption. First, some high-performance carriers have uncleared long-term safety. Examples include MOFs and TiO_2_ nanoparticles, whose migration behavior has not been fully studied [159,160,161,162]. Additionally, complex synthesis and high costs limit large-scale production. Second, most responsive systems are validated under simplified, static conditions, whereas real food supply chains involve dynamic fluctuations in temperature, humidity, and gas composition that may compromise release precision. Third, a gap persists between laboratory evaluation and practical application: limited attention has been paid to carrier-food matrix interactions, sensory impacts, and regulation of mixed microbial communities in complex systems, constraining real-world efficacy [158,163,164,165].

Although these limitations are substantial, they do not diminish the conceptual value of stimulus-responsive strategies. To address these challenges, future development should prioritize the following directions:

(1) Multifunctional integration and intelligent upgrading. Packaging systems with only indicative or single-stimulus responsiveness are insufficient for complex preservation demands. Hence, integrating sensing capabilities into carriers, such as developing composite systems for simultaneous release and fluorescence detection or constructing closed-loop systems for environmental monitoring–response regulation–efficacy feedback, is essential for advancing packaging intelligence.
(2) Green sustainability and scalable production. Emphasis should be placed on biobased, degradable carriers and solvent-free synthesis processes to reduce costs and environmental footprint, facilitating industrial translation.
(3) Enhanced adaptability in complex systems. Leveraging multi-omics technologies to understand food-carrier interaction mechanisms and designing multi-stimuli-responsive units will improve release precision under fluctuating conditions.
(4) Standardized safety and assessment systems. Developing unified evaluation criteria for carrier biocompatibility, including long-term in vivo toxicity and bioactive migration, will help bridge laboratory findings with regulatory requirements for industrial applications.
(5) Cross-disciplinary collaborative innovation. Given the intricate interplay between material performance, microbial dynamics, and industrial scalability, interdisciplinary collaboration among materials science, food microbiology, and process engineering is crucial. For example, metaproteomics can inform the rational design of enzyme-responsive carriers by identifying key enzymes in spoilage microbial communities. In turn, process engineering can optimize the scale-up of advanced materials by adjusting homogenization parameters for MOF-based hydrogels or microfluidic parameters for lipid nanocarriers, ensuring both performance retention and industrial feasibility.

## 6. Conclusions

CRFP represents a transformative paradigm in food preservation, evolving from passive barrier functions toward adaptive, intelligence-driven systems capable of sensing and responding to dynamic microenvironmental cues. This review systematically examines recent advances in single- and multi-stimulus-responsive systems. It clarifies the design principles and functional performance of stimuli-responsive materials and encapsulation technologies. Particular attention has been given to the rational integration of carrier architectures with triggerable release behaviors tailored to specific spoilage pathways. Despite these advances, critical challenges remain regarding long-term biosafety, regulatory acceptance, scalable manufacturing, and reliable performance under realistic, fluctuating supply chain conditions. Looking forward, future research should prioritize the following directions to accelerate the translation of CRFP from laboratory concepts to practical applications:

First, developing bio-based and biodegradable responsive materials with proven safety profiles. Given the regulatory and safety uncertainties surrounding advanced carriers such as MOFs and metal oxide nanoparticles, efforts should focus on naturally derived alternatives (e.g., chitosan, starch, cyclodextrins) and food-grade synthetic polymers that already possess regulatory clearance. Research should also establish standardized migration testing protocols specifically tailored for stimulus-responsive systems.

Second, designing multifunctional systems that integrate sensing, response, and feedback capabilities. Future work should explore architectures where the release of bioactives is dynamically adjusted based on real-time spoilage indicators. This could be achieved through the incorporation of embedded sensors or the use of multi-stimulus-responsive platforms that allow sequential or synergistic control.

Third, enhancing system robustness under real-world conditions. Most studies validate performance under idealized, static conditions. Future research should systematically evaluate responsive packaging under dynamic temperature, humidity, and microbial fluctuations that mimic actual supply chains.

Fourth, establishing standardized evaluation frameworks. This includes developing consensus on performance metrics and creating food-simulant models that accurately represent the buffering and interference effects of real food matrices. Collaboration between materials scientists, food microbiologists, and regulatory experts will be essential to bridge the gap between laboratory breakthroughs and commercial deployment.

Continued progress in these directions will position CRFP to make meaningful contributions toward more sustainable, efficient, and intelligent food preservation systems.

## Figures and Tables

**Table 1:** Corrective responsive systems for food preservation.

Type	Stimulus	Functional Material/System	Functions	Key Quantitative Metrics	References
Single-stimulus	pH	Polyvinyl alcohol/poly(acrylic acid) (PVA/PAA) composite matrix loaded with aminoethyl-phloretin (AEP)	pH-responsive release; antioxidant and antibacterial activity; pork preservation	AEP release: ~90% (pH 9, 60 h), ~80% (pH 2, 60 h)	[16]
		Chitosan matrix with alizarin	pH-responsive color change; antioxidant and antibacterial activity	Alizarin release: 13 μg·gmm^−2^ (90 min, 50% ethanol); Color change: pH 4 (yellow) → pH 10 (purple); ammonia response time: 4 min, S_RGB_ = 9.3%	[19]
		Poly(D,L-lactide-co-glycolide) (PLGA)/chitosan nanoparticles loaded with trans-cinnamaldehyde (TCIN)	pH-responsive controlled release; antimicrobial activity	Initial burst release: ~60% (pH 4, 2 h)	[20]
		Aldehyde-grafted chitosan films via reversible imine bonds	pH-responsive release; antifungal activity	Headspace benzaldehyde: 414 ng·mL^−1^·g_film_^−1^ (pH 4, 7 d) and 203 ng·mL^−1^·g_film_^−1^ (pH 7, 7 d)	[24]
		Chitosan grafted with trans-2-hexenal (HX) and salicylaldehyde (SL)	pH-responsive release; antimicrobial activity; fresh-cut pineapple preservation	Release at pH 3 (24 h): SL 9.3 mg·L^−1^·g_film_^−1^, HX 5.0 mg·L^−1^·g_film_^−1^	[25]
	Enzyme	Poly(butylene adipate terephthalate) (PBAT)/zein composite fibers loaded with thymol	Enzyme-responsive release; antioxidant and antibacterial activity; chilled mutton preservation	Enzyme-triggered release: >99% (3 h)	[33]
		Azobenzene-containing polyurethane polymersomes	Enzyme-triggered degradation; controlled bioactive release	Degradation: 12 h (azoreductase); Doxorubicin release: 65% (48 h)	[34]
	Relative humidity (RH)	Curcumin (Cur)-loaded HKUST-1 grown in carboxymethyl starch (CMS)/PVA matrix	Moisture-responsive release; antioxidant and antibacterial activity; fruit preservation	Release: 58.3% (30% moisture, 168 h) vs. 25.1% (0% moisture)	[46]
		Poly(Ethylene Glycol)-Poly(ɛ-Caprolactone) (PEG-PCL) nanomicelles loaded with cinnamon essential oil (CEO)	Humidity-controlled release; antifungal activity; strawberry preservation	CEO release: <30% (15% RH), <35% (35% RH), ~72% (75% RH) at 168 h	[47]
		CEO-micelles combined with 1-methylcyclopropene/cyclodextrin (1-MCP/α-CD) in PVA nanofiber film	Synergistic preservation; delayed senescence, enhanced antioxidant capacity	Release: ~72% (75% RH, 168 h)	[48]
		Carboxymethyl cellulose (CMC)/zein film with pterostilbene@ β-cyclodextrin inclusion complexes	Humidity-responsive release; antimicrobial and antioxidant activities; fruit preservation	PTE release: 55.4% (24 h, 98% RH) → 89.0% (144 h, 98% RH); at 49% RH only 22.7%	[50]
		The triple-layer electrospun pad comprising a cellulose acetate (CA) transfer layer, a NaClO_2_-loaded PVA-g-PAA absorbent layer, and a polyurethane barrier layer	Directional liquid absorption; Humidity-controlled ClO_2_ release; Broad-spectrum antimicrobial activity; Strawberry preservation	ClO_2_ release (4 d): 90.45% (90% RH), 62.75% (60% RH), 26.48% (30% RH)	[53]
		CA film loaded with ground mustard seeds	Moisture-responsive allyl isothiocyanate (AITC) release; antimicrobial activity; beef preservation	Higher release at 100% RH vs. 75.5% RH; Max headspace AITC (4 d): 12 μg·L^−1^ (low-fat ground beef, 9% fat), 6 μg·L^−1^ (medium-fat ground beef, 19% fat)	[54]
	Temperature	Lemon essential oil (LEO)-loaded polylactic acid (PLA) nanofibers with poly(N-isopropylacrylamide) (PNIPAAm) thermosensitive layer	Thermo-controlled LEO release; antimicrobial and antioxidant activities; blackberry preservation	LEO release (24 h): PLA/LEO 45.49 ± 0.88% (20 °C), PLA/LEO 85.33 ± 0.19% (40 °C), PLP 6.15 ± 0.01% (20 °C), PLP 53.69 ± 0.56% (40 °C)	[67]
		PAA-g-β-CD/PAAm IPN hydrogel	Thermoresponsive behavior; controlled drug release	Ibuprofen release (12 h): IPN 79.4% (37 °C) and 25.2% (25 °C); PAA/PAAm 84.2% (37 °C) and 28.4% (25 °C)	[69]
	Light	Azobenzene-functionalized shell with basil/thyme essential oils (EOs) core	Ultraviolet (UV)-induced EOs release	UV light: 360 nm, 5.5 W/m^2^; EO release at 180 min: Nanocapsule (NC)B 60% (continuous), 49% (pulsed); NCT 20% (continuous), 20% (pulsed); 63% released from NCB within 30 min	[79]
		PE or PLA films coated with polyamide nanocapsules loaded with thyme EOs	Light-triggered EOs release; antimicrobial activity; non-contact preservation	UV light: 365 nm, 15 min; Thymol headspace: 8-fold increase at 24 h post-irradiation	[80]
		Nanoparticles (NPs) with quinone-methide self-immolative moiety and light-sensitive groups in the backbone, loaded with Nile Red	Light-responsive Nile Red release	UV 350 nm: complete deprotection (15 min); Near-infrared (NIR) 750 nm: equivalent degradation (5 h); Nile Red burst release (1 min UV), sustained release (4 h NIR)	[82]
		PLGA hollow microsphere cores loaded with Van and PPy NPs	Photothermal response; controlled drug release; bactericidal effect	Trigger light: 808 nm NIR, 0.5 W·cm^−2^, 15 min; Photothermal effect: temperature rose to ~60 °C within 5 min in vitro and ~50 °C in vivo after 15 min; Drug release: Van release exceeds therapeutic threshold within 15 min	[85]
		Pullulan (PUL)/PVA nanofibers with thymol (THY)-loaded PCN-224	Controlled thymol release; antimicrobial activity; fruit preservation	Singlet oxygen detection: 43.52% DPBF decrease under (532 nm, 30 min); antibacterial rate: ~99% against *E. coli* and ~98% against *S. aureus*	[73]
		KC/Cur-OSAS (K-carrageenan with curcumin and octenyl succinic anhydride starch) composite coating	Photodynamic antibacterial and antioxidant capacity; grape preservation	Antibacterial efficacy: >99% (*S. aureus* and *E. coli*, 15 min blue light)	[74]
		Chitosan matrix with TiO_2_ nanoparticles	Ethylene photodegradation; antimicrobial activity	Ethylene degradation: ~12% (CT1, 180 min UV); antibacterial rate (CT1 + UV): ~50% for *S. aureus*, ~40% for *E. coli*, ~30% for fungi	[75]
	Glutathione	SO_2_-releasing dendronized chitooligosaccharide	Glutathione-responsive SO_2_ release; antibacterial activity	SO_2_ release: ~0.83 pM per 1 mg·mL^−1^ Cos-G3-Dns (200 μM glutathione responsive)	[96]
	Phosphate	HKUST-1@carboxymethyl chitosan (HKUST-1@CMCS)	Phosphate-responsive release; antimicrobial activity; strawberry preservation	Dimethyl fumarate release (84 h): 28.6% (water), 57.6% (0.01 M PBS), 66.8% (0.02 M PBS), 75.4% (0.04 M PBS); maximum release duration: 384 h	[97]
Multi-stimulus	pH/enzyme dual-responsive	Carvacrol/zeolitic imidazolate framework-8/TEMPO-oxidized cellulose nanofibers/pectin (CAR@ZIF-8/TOCNF/Pec) composite film	Dual-responsive controlled release; antimicrobial activity; fruit preservation	Carvacrol release at 168 h: 86.84% (pH 5.0), 88.65% (1.0 mg·mL^−1^ pectinase), 49.80% (pH 7.0), 24.17% (pH 9.0)	[35]
	pH/temperature dual-responsive	PVA/PAA IPN hydrogels	Dual-responsive drug release	Pulsatile release pattern, complete release within 25 h; higher release at 45 °C (pH 7, “on”) vs. 25 °C (“off”); reversible oscillatory release between pH 4 and 7	[100]
	Enzymes (protease, amylase, cellulase) and RH	Cellulose/zein/starch fibers with antimicrobials and cyclodextrin-inclusion complexes	Enzyme/RH-responsive release; antimicrobial activity	Enzyme-triggered release (12 h): 23% (1 U·mL^−1^), 13% (PBS); complete degradation at 3 U·mL^−1^; RH-responsive: thymol ~7.5 ppm at 95% RH and ~11.5 ppm at 50% RH; CD-IC dissociated at RH > 85%	[101]
	pH and Enzyme (glucoamylase)	THY@PLA-COS-DAS (THY-loaded PLA nanofibers modified with chitosan oligosaccharide and dialdehyde starch)	pH/Amylase-responsive thymol release; antifungal activity	Thymol release (120 h): 78.1% (pH 5.0), 98.6% (2.0 mg/mL glucoamylase), 42.3% (pH 7.0)	[98]
	pH and RH	LA@Cu-MOF film combined with sodium alginate	pH/RH-responsive α-LA release; antimicrobial activity; fruit preservation	α-LA release (48 h): 76.50% (90% RH), 22.16% (30% RH); release concentration (48 h): 218.46 mg·L^−1^ (pH 5.0), 201.39 mg·L^−1^ (pH 6.0), 101.90 mg·L^−1^ (pH 7.0)	[99]

**Table 2:** Material maturity and translational readiness in CRFP.

Material Category	Representative Examples	Technology Readiness Level (Estimated)	Regulatory Risk	Scalability	Cost Level	Overall Translational Potential
Natural biopolymers	Chitosan, starch derivatives	Medium–High	Low	High	Moderate	High
Synthetic food-contact polymers	PEG-based, polyesters	Medium–High	Low–Medium	High	Low–Moderate	High
Metal–Organic Frameworks (MOFs)	Zn-, Cu-based MOFs	Low–Medium	High	Low	High	Limited
Metal oxide nanoparticles	TiO_2_, ZnO	Medium	Medium–High	Moderate	Moderate–High	Conditional
Photoresponsive nanocomposites	Azobenzene systems, photocatalytic hybrids	Low	High	Low	High	Limited

**Table 3:** CRFP design matrix.

Food Spoilage Type	Optimal Stimulus	Preferred Carrier	Recommended Release Kinetics
Microbial spoilage	pH; total volatile basic nitrogen (TVB-N)	Chitosan-based films; microencapsulated essential oils	Rapid initial release followed by sustained diffusion
Lipid oxidation	Oxygen exposure; light	antioxidant-loaded microcapsules	Gradual, long-term controlled release
Moisture-induced spoilage	RH increase	Hydrophilic biopolymer networks; humidity-responsive hydrogels	Moisture-triggered burst release
Temperature abuse	Temperature deviation	Thermoresponsive polymer matrices	Temperature-responsive on-demand release
Multi-factor spoilage systems	Multiple triggers	Multilayer structured films; hybrid composites	Sequential or staged release

## Data Availability

Data sharing is not applicable.

## Abbreviations

The following abbreviations are used in this manuscript:

CRFP	Corrective Responsive Food Packaging
ABTS	2,2′-Azino-Bis(3-Ethylbenzothiazoline-6-Sulfonic Acid)
AITC	Allyl Isothiocyanate
CA	cellulose acetate
CMC	Carboxymethyl Cellulose
CMCS	Carboxymethyl Chitosan
CNCs	Cellulose Nanocrystals
Cur	Curcumin
DAS	Dialdehyde Starch
DPPH	2,2-Diphenyl-1-Picrylhydrazyl
EC	Ethylcellulose
EO	Essential Oil
FAO	Food and Agriculture Organization of the United Nations
GRAS	Generally Recognized As Safe
HKUST-1	MOF-199
LCST	Low Critical Solution Temperature
UCST	Upper Critical Solution Temperature
LEO	Lemon Essential Oil
MOFs	Metal–Organic Frameworks
MSNs	Mesoporous Silica Nanoparticles
NLCs	Nanostructured Lipid Carriers
NPs	Nanoparticles
OSA	Octenyl Succinic Anhydride
PAA	Poly(Acrylic Acid)
PBAT	Poly(Butylene Adipate Terephthalate)
PDMA	Poly(Dimethylaminoethyl Methacrylate)
PDMAAm	Poly(N,N-Dimethylacrylamide)
PEG-PCL	Poly(Ethylene Glycol)-Poly(ɛ-Caprolactone)
PEO	Poly(Ethylene Oxide)
PLA	Polylactic Acid
PLGA	Poly(D,L-Lactide-Co-Glycolide)
PNIPAAm	Poly(N-Isopropylacrylamide)
PBS	Phosphate-Buffered Saline
PCL	Poly(ɛ-Caprolactone)
PTE	Pterostilbene
PVA	Polyvinyl Alcohol
RH	Relative Humidity
ROS	Reactive Oxygen Species
RFP	Responsive Food Packaging
*E. coli*	*Escherichia coli*
*R. stolonifer*	*Rhizopus stolonifer*
*S. aureus*	*Staphylococcus aureus*
*S. typhimurium*	*Salmonella typhimurium*
*P. aeruginosa*	*Pseudomonas aeruginosa*
*B. cinerea*	*Botrytis cinerea*
*A. oryzae*	*Aspergillus oryzae*
*P. roqueforti*	*Penicillium roqueforti*
SA	Sodium Alginate
SLNs	Solid Lipid Nanoparticles
TCIN	Trans-Cinnamaldehyde
THY	Thymol
TiO_2_	Titanium Dioxide
TOCNF	TEMPO-Oxidized Cellulose Nanofibers
UV	Ultraviolet
TVB-N	Total Volatile Basic Nitrogen
CAR	Carvacrol
CEO	Cinnamon Essential Oil
1-MCP	1-Methylcyclopropene
ZIF-8	Zeolitic Imidazolate Framework-8
ZnO	Zinc Oxide
